# Supplementary material for: A Likelihood Approach for Real-Time Calibration of Stochastic Compartmental Epidemic Models
Source: PLoS Comput Biol. 2017 Jan 17;13(1):e1005257. doi: 10.1371/journal.pcbi.1005257 (PMC5240920; doi:10.1371/journal.pcbi.1005257)
Supplement: S1 File — (TAR.GZ) [file pcbi.1005257.s014.tar.gz › HSPH_Online-SI-Revision/IPoi09/n10-severe/n10-severe_BENCH2_table4.pdf]

1	newly 3 weeks spec	197.	234.508	{111., 420.}	49.7059	0.179492	newly 3 weeks cumul	743.	895.365	{443., 1552.}	47.2452	0.168113	newly 5 weeks spec	99.	145.365	{68., 292.}	63.7005	0.183237	newly 5 weeks cumul	972.	1228.27	{598., 2149.}	50.5177	0.170054	newly 47 weeks cumul	1325.	1726.24	{833., 3028.}	54.0665	0.175567
2	newly 3 weeks spec	183.	441.667	{249., 763.}	141.348	0.344781	newly 3 weeks cumul	725.	1558.75	{927., 2475.}	114.999	0.308149	newly 5 weeks spec	116.	296.571	{149., 508.}	155.665	0.35322	newly 5 weeks cumul	1003.	2219.6	{1258., 3623.}	121.296	0.313862	newly 49 weeks cumul	1262.	3284.98	{1760., 5456.}	160.3	0.373066
3	newly 3 weeks spec	87.	259.945	{148., 396.}	198.788	0.43797	newly 3 weeks cumul	412.	1045.38	{631., 1545.}	153.734	0.371466	newly 5 weeks spec	40.	145.479	{80., 228.}	263.699	0.516527	newly 5 weeks cumul	486.	1386.04	{820., 2044.}	185.194	0.420401	newly 51 weeks cumul	514.	1788.4	{1050., 2663.}	247.937	0.504624
4	newly 3 weeks spec	158.	266.155	{122., 482.}	82.8787	0.235106	newly 3 weeks cumul	634.	965.862	{494., 1654.}	66.4201	0.204979	newly 5 weeks spec	66.	188.69	{71., 335.}	188.61	0.371156	newly 5 weeks cumul	805.	1383.55	{664., 2433.}	82.6601	0.231307	newly 46 weeks cumul	946.	2188.69	{906., 3843.}	135.985	0.29918
5	newly 3 weeks spec	156.	266.155	{122., 482.}	84.3833	0.237014	newly 3 weeks cumul	639.	965.862	{494., 1654.}	65.6306	0.203803	newly 5 weeks spec	112.	188.69	{71., 335.}	89.5628	0.243314	newly 5 weeks cumul	896.	1383.55	{664., 2433.}	70.8321	0.21555	newly 46 weeks cumul	1167.	2188.69	{906., 3843.}	100.405	0.251479
6	newly 3 weeks spec	63.	380.931	{198., 618.}	504.652	0.736211	newly 3 weeks cumul	405.	1328.62	{772., 2024.}	228.582	0.482242	newly 5 weeks spec	51.	266.155	{122., 482.}	421.873	0.651193	newly 5 weeks cumul	521.	1913.55	{1061., 3079.}	267.284	0.526783	newly 47 weeks cumul	643.	3136.38	{1483., 5365.}	387.773	0.626906
7	newly 3 weeks spec	115.	242.338	{97., 548.}	118.274	0.270225	newly 3 weeks cumul	383.	887.338	{421., 1804.}	134.694	0.31357	newly 5 weeks spec	76.	168.015	{55., 361.}	131.154	0.261925	newly 5 weeks cumul	548.	1260.09	{562., 2826.}	133.689	0.301166	newly 46 weeks cumul	668.	1878.17	{734., 3925.}	183.871	0.353556
8	newly 3 weeks spec	100.	310.328	{161., 597.}	210.328	0.431751	newly 3 weeks cumul	467.	1182.75	{657., 2218.}	153.266	0.359399	newly 5 weeks spec	34.	186.656	{92., 373.}	448.987	0.66712	newly 5 weeks cumul	561.	1611.33	{868., 3119.}	187.224	0.408188	newly 50 weeks cumul	610.	2188.02	{1139., 4366.}	258.691	0.496759
9	newly 3 weeks spec	135.	362.919	{188., 600.}	169.092	0.368989	newly 3 weeks cumul	764.	1461.42	{842., 2411.}	92.6237	0.246454	newly 5 weeks spec	57.	190.355	{91., 277.}	233.956	0.450048	newly 5 weeks cumul	905.	1919.16	{1082., 3094.}	112.497	0.28123	newly 54 weeks cumul	1026.	2389.89	{1293., 3898.}	132.992	0.313471
10	newly 3 weeks spec	69.	190.984	{104., 315.}	176.788	0.385868	newly 3 weeks cumul	417.	870.581	{471., 1426.}	109.004	0.269798	newly 5 weeks spec	31.	89.3871	{42., 160.}	189.074	0.390402	newly 5 weeks cumul	500.	1090.24	{597., 1782.}	118.216	0.285314	newly 54 weeks cumul	536.	1266.5	{675., 2042.}	136.287	0.317866
11	newly 3 weeks spec	22.	265.2	{105., 504.}	1105.45	1.00484	newly 3 weeks cumul	176.	1138.4	{493., 2216.}	546.818	0.748701	newly 5 weeks spec	7.	137.815	{53., 269.}	1868.79	1.20433	newly 5 weeks cumul	192.	1466.52	{615., 2874.}	663.814	0.816712	newly 53 weeks cumul	192.	1779.12	{732., 3470.}	826.627	0.896761
12	newly 3 weeks spec	117.	421.109	{215., 549.}	259.923	0.507927	newly 3 weeks cumul	568.	1620.72	{933., 2147.}	185.338	0.424946	newly 5 weeks spec	54.	243.641	{133., 329.}	351.186	0.588506	newly 5 weeks cumul	704.	2189.11	{1212., 2865.}	210.953	0.455728	newly 53 weeks cumul	804.	2845.97	{1499., 3704.}	253.976	0.50411
13	newly 3 weeks spec	134.	296.82	{153., 460.}	122.51	0.292787	newly 3 weeks cumul	648.	1124.11	{653., 1673.}	76.5559	0.213107	newly 5 weeks spec	83.	185.131	{91., 297.}	125.025	0.289421	newly 5 weeks cumul	810.	1543.38	{878., 2344.}	92.6655	0.242818	newly 50 weeks cumul	934.	2145.54	{1184., 3286.}	130.014	0.306507
14	newly 3 weeks spec	60.	247.887	{123., 437.}	313.145	0.559382	newly 3 weeks cumul	328.	1044.95	{525., 1777.}	218.583	0.451718	newly 5 weeks spec	20.	130.226	{61., 218.}	551.129	0.7424	newly 5 weeks cumul	376.	1353.71	{673., 2289.}	260.029	0.501441	newly 52 weeks cumul	397.	1656.94	{804., 2798.}	317.364	0.562659
15	newly 3 weeks spec	54.	153.328	{87., 267.}	183.94	0.400959	newly 3 weeks cumul	371.	780.787	{440., 1350.}	111.029	0.279202	newly 5 weeks spec	11.	57.7869	{29., 98.}	425.335	0.653236	newly 5 weeks cumul	406.	935.18	{540., 1607.}	130.429	0.314949	newly 56 weeks cumul	423.	1030.08	{580., 1783.}	143.518	0.337842
16	newly 3 weeks spec	69.	137.371	{64., 227.}	103.296	0.235031	newly 3 weeks cumul	368.	595.113	{288., 910.}	70.5075	0.180128	newly 5 weeks spec	29.	69.9194	{30., 104.}	144.105	0.305305	newly 5 weeks cumul	440.	763.387	{378., 1178.}	79.7214	0.194129	newly 52 weeks cumul	501.	928.468	{434., 1487.}	90.3	0.214243
17	newly 3 weeks spec	221.	287.2	{153., 472.}	47.4695	0.154838	newly 3 weeks cumul	873.	1108.12	{623., 1768.}	42.0707	0.144286	newly 5 weeks spec	183.	168.877	{81., 238.}	43.2114	0.19006	newly 5 weeks cumul	1256.	1494.71	{809., 2395.}	39.7501	0.141996	newly 52 weeks cumul	1842.	1992.45	{1024., 3169.}	38.7296	0.148535
18	newly 3 weeks spec	189.	367.459	{217., 547.}	96.5218	0.255782	newly 3 weeks cumul	833.	1368.21	{864., 2077.}	67.156	0.199098	newly 5 weeks spec	133.	234.131	{113., 374.}	82.5958	0.211704	newly 5 weeks cumul	1151.	1899.16	{1146., 2911.}	68.6702	0.197873	newly 50 weeks cumul	1580.	2686.46	{1518., 4150.}	74.0921	0.202518
19	newly 3 weeks spec	134.	318.776	{157., 573.}	139.488	0.332185	newly 3 weeks cumul	645.	1142.28	{621., 1870.}	82.0583	0.231765	newly 5 weeks spec	78.	229.	{90., 409.}	195.314	0.390794	newly 5 weeks cumul	830.	1637.43	{845., 2707.}	100.704	0.265377	newly 46 weeks cumul	1044.	2631.26	{1176., 4593.}	154.329	0.338593
20	newly 3 weeks spec	271.	324.255	{175., 529.}	37.7256	0.1482	newly 3 weeks cumul	966.	1143.07	{664., 1811.}	36.0305	0.147494	newly 5 weeks spec	160.	218.345	{119., 373.}	48.8523	0.159831	newly 5 weeks cumul	1369.	1625.69	{940., 2630.}	35.5774	0.138595	newly 46 weeks cumul	1954.	2727.6	{1363., 4255.}	52.6305	0.165621
21	newly 3 weeks spec	92.	216.984	{107., 403.}	135.992	0.307727	newly 3 weeks cumul	451.	976.742	{504., 1791.}	116.623	0.279597	newly 5 weeks spec	39.	103.274	{45., 190.}	164.806	0.344511	newly 5 weeks cumul	575.	1229.9	{631., 2258.}	114.042	0.271983	newly 54 weeks cumul	717.	1444.58	{725., 2673.}	102.672	0.248454
22	newly 3 weeks spec	126.	359.015	{169., 697.}	185.236	0.397969	newly 3 weeks cumul	547.	1393.96	{723., 2512.}	155.143	0.367041	newly 5 weeks spec	54.	201.765	{79., 396.}	273.638	0.489195	newly 5 weeks cumul	674.	1867.53	{935., 3456.}	177.269	0.39515	newly 52 weeks cumul	743.	2387.	{1141., 4473.}	221.265	0.44883
23	newly 3 weeks spec	180.	231.828	{92., 391.}	59.4184	0.196916	newly 3 weeks cumul	675.	935.109	{376., 1493.}	60.3773	0.189607	newly 5 weeks spec	110.	126.453	{47., 199.}	57.5426	0.216622	newly 5 weeks cumul	921.	1232.39	{497., 1948.}	59.5905	0.192311	newly 51 weeks cumul	1186.	1564.33	{620., 2500.}	60.2616	0.195555
24	newly 3 weeks spec	117.	449.115	{248., 854.}	283.859	0.539784	newly 3 weeks cumul	561.	1662.54	{981., 2861.}	196.353	0.443991	newly 5 weeks spec	36.	279.393	{144., 572.}	676.093	0.826819	newly 5 weeks cumul	658.	2299.74	{1330., 4198.}	249.504	0.508271	newly 52 weeks cumul	737.	3168.15	{1714., 6049.}	329.871	0.587716
25	newly 3 weeks spec	33.	306.754	{151., 648.}	829.558	0.898272	newly 3 weeks cumul	182.	1342.36	{649., 2545.}	637.561	0.812618	newly 5 weeks spec	2.	143.279	{65., 276.}	7063.93	1.77297	newly 5 weeks cumul	198.	1693.2	{823., 3232.}	755.15	0.87281	newly 55 weeks cumul	198.	1976.49	{917., 3747.}	898.228	0.936436
26	newly 3 weeks spec	84.	190.	{88., 324.}	128.798	0.287076	newly 3 weeks cumul	510.	902.19	{426., 1486.}	83.7224	0.21357	newly 5 weeks spec	55.	82.1111	{38., 129.}	70.4185	0.19407	newly 5 weeks cumul	628.	1111.62	{524., 1821.}	84.2534	0.21248	newly 54 weeks cumul	701.	1261.57	{580., 2074.}	87.0367	0.216388
27	newly 3 weeks spec	183.	312.516	{162., 569.}	76.0797	0.200232	newly 3 weeks cumul	804.	1363.06	{726., 2448.}	73.359	0.201095	newly 5 weeks spec	88.	149.887	{71., 277.}	77.4377	0.197755	newly 5 weeks cumul	1035.	1729.94	{929., 3128.}	71.9526	0.195929	newly 55 weeks cumul	1231.	2047.89	{1059., 3733.}	72.1325	0.195105
28	newly 3 weeks spec	70.	302.639	{143., 554.}	332.342	0.569732	newly 3 weeks cumul	412.	1296.84	{662., 2323.}	214.766	0.446011	newly 5 weeks spec	15.	151.951	{66., 253.}	913.005	0.928503	newly 5 weeks cumul	458.	1663.3	{832., 2975.}	263.165	0.504277	newly 54 weeks cumul	486.	1999.64	{987., 3615.}	311.448	0.554578
29	newly 3 weeks spec	27.	122.206	{55., 179.}	352.616	0.586562	newly 3 weeks cumul	254.	649.	{329., 940.}	155.674	0.35135	newly 5 weeks spec	11.	44.0159	{20., 64.}	301.587	0.531299	newly 5 weeks cumul	278.	766.317	{386., 1120.}	175.654	0.381936	newly 56 weeks cumul	279.	832.46	{413., 1225.}	198.373	0.415587
30	newly 3 weeks spec	46.	236.581	{123., 406.}	414.306	0.656935	newly 3 weeks cumul	317.	1119.92	{594., 1821.}	253.287	0.502173	newly 5 weeks spec	3.	101.306	{46., 174.}	3276.88	1.46359	newly 5 weeks cumul	342.	1377.44	{716., 2261.}	302.759	0.556655	newly 56 weeks cumul	343.	1559.21	{804., 2566.}	354.58	0.607437
31	newly 3 weeks spec	146.	144.321	{49., 267.}	60.3718	0.268213	newly 3 weeks cumul	569.	672.821	{267., 1198.}	58.009	0.215598	newly 5 weeks spec	41.	61.9286	{18., 112.}	85.8885	0.253875	newly 5 weeks cumul	686.	833.304	{318., 1496.}	60.9668	0.218806	newly 53 weeks cumul	786.	949.054	{349., 1693.}	61.848	0.222647
32	newly 3 weeks spec	142.	206.807	{79., 392.}	67.4821	0.214092	newly 3 weeks cumul	533.	779.737	{318., 1339.}	64.7971	0.210413	newly 5 weeks spec	69.	132.754	{47., 257.}	107.958	0.26717	newly 5 weeks cumul	702.	1078.82	{427., 1895.}	71.5924	0.220309	newly 46 weeks cumul	971.	1521.3	{571., 2751.}	75.5181	0.220678
33	newly 3 weeks spec	184.	266.155	{122., 482.}	66.8759	0.214921	newly 3 weeks cumul	700.	965.862	{494., 1654.}	57.3153	0.191988	newly 5 weeks spec	80.	188.69	{71., 335.}	141.853	0.305341	newly 5 weeks cumul	923.	1383.55	{664., 2433.}	67.9232	0.21186	newly 46 weeks cumul	1232.	2188.69	{906., 3843.}	93.2462	0.243171
34	newly 3 weeks spec	186.	266.633	{115., 456.}	60.6272	0.188478	newly 3 weeks cumul	603.	991.417	{456., 1679.}	73.1979	0.209596	newly 5 weeks spec	102.	178.65	{71., 309.}	87.9902	0.228794	newly 5 weeks cumul	831.	1391.7	{633., 2353.}	76.3377	0.214166	newly 46 weeks cumul	1130.	2103.57	{872., 3422.}	94.5339	0.237297
35	newly 3 weeks spec	127.	231.58	{101., 421.}	89.7638	0.231337	newly 3 weeks cumul	520.	984.391	{471., 1756.}	94.0719	0.243667	newly 5 weeks spec	58.	120.971	{49., 220.}	116.117	0.273525	newly 5 weeks cumul	660.	1275.33	{580., 2246.}	98.3004	0.249906	newly 53 weeks cumul	785.	1560.1	{701., 2725.}	103.303	0.256366
36	newly 3 weeks spec	227.	364.286	{182., 652.}	68.5197	0.193046	newly 3 weeks cumul	850.	1323.62	{750., 2221.}	61.1018	0.182214	newly 5 weeks spec	131.	234.508	{111., 420.}	86.1626	0.22443	newly 5 weeks cumul	1137.	1854.7	{982., 3121.}	68.0474	0.194733	newly 49 weeks cumul	1502.	2685.57	{1401., 4547.}	82.3843	0.218591
37	newly 3 weeks spec	162.	348.984	{171., 621.}	117.656	0.281566	newly 3 weeks cumul	656.	1359.14	{723., 2444.}	109.122	0.28265	newly 5 weeks spec	79.	198.016	{85., 365.}	152.542	0.316535	newly 5 weeks cumul	838.	1826.02	{922., 3285.}	119.837	0.295626	newly 52 weeks cumul	1008.	2338.	{1098., 4119.}	133.582	0.310262
38	newly 3 weeks spec	199.	394.355	{198., 655.}	100.259	0.255822	newly 3 weeks cumul	840.	1502.34	{830., 2355.}	80.3936	0.223776	newly 5 weeks spec	85.	230.21	{115., 360.}	170.835	0.371806	newly 5 weeks cumul	1088.	2038.9	{1114., 3156.}	88.6741	0.237885	newly 52 weeks cumul	1378.	2711.74	{1402., 4219.}	97.7223	0.250402
39	newly 3 weeks spec	207.	364.286	{182., 652.}	80.7837	0.216472	newly 3 weeks cumul	748.	1323.62	{750., 2221.}	78.5417	0.219416	newly 5 weeks spec	130.	234.508	{111., 420.}	87.2772	0.226384	newly 5 weeks cumul	1035.	1854.7	{982., 3121.}	81.4523	0.222529	newly 48 weeks cumul	1546.	2685.57	{1401., 4547.}	78.1505	0.210263
40	newly 3 weeks spec	211.	296.571	{149., 508.}	55.691	0.171465	newly 3 weeks cumul	819.	1102.52	{573., 1908.}	49.8324	0.160802	newly 5 weeks spec	116.	187.54	{84., 363.}	72.6464	0.193452	newly 5 weeks cumul	1057.	1524.57	{760., 2678.}	56.9581	0.172326	newly 48 weeks cumul	1430.	2167.9	{1083., 3741.}	63.5187	0.182392
41	newly 3 weeks spec	77.	227.048	{111., 346.}	194.868	0.398316	newly 3 weeks cumul	495.	1061.23	{536., 1583.}	115.497	0.27902	newly 5 weeks spec	16.	96.1452	{39., 144.}	500.907	0.683902	newly 5 weeks cumul	559.	1304.53	{636., 1948.}	133.727	0.308076	newly 56 weeks cumul	602.	1473.56	{697., 2214.}	144.923	0.325162
42	newly 3 weeks spec	107.	380.931	{198., 618.}	256.01	0.506167	newly 3 weeks cumul	500.	1328.62	{772., 2024.}	166.807	0.393883	newly 5 weeks spec	44.	266.155	{122., 482.}	504.898	0.715311	newly 5 weeks cumul	615.	1913.55	{1061., 3079.}	211.147	0.454745	newly 47 weeks cumul	696.	3136.38	{1483., 5365.}	350.629	0.592508
43	newly 3 weeks spec	71.	161.017	{68., 304.}	130.446	0.295856	newly 3 weeks cumul	380.	781.417	{365., 1452.}	109.478	0.264268	newly 5 weeks spec	40.	67.05	{33., 129.}	77.125	0.193626	newly 5 weeks cumul	453.	950.583	{435., 1765.}	113.293	0.268902	newly 55 weeks cumul	496.	1060.08	{468., 1970.}	117.134	0.274628
44	newly 3 weeks spec	171.	344.677	{154., 695.}	106.735	0.263054	newly 3 weeks cumul	617.	1256.74	{643., 2166.}	105.375	0.274466	newly 5 weeks spec	118.	230.113	{99., 417.}	102.993	0.23667	newly 5 weeks cumul	869.	1772.79	{865., 3345.}	106.413	0.268476	newly 49 weeks cumul	1172.	2538.58	{1142., 4873.}	119.556	0.278952
45	newly 3 weeks spec	123.	328.	{165., 577.}	166.667	0.372947	newly 3 weeks cumul	569.	1287.84	{684., 2170.}	126.334	0.313429	newly 5 weeks spec	45.	183.516	{87., 288.}	307.814	0.543769	newly 5 weeks cumul	691.	1715.56	{905., 2925.}	148.273	0.348882	newly 52 weeks cumul	789.	2217.61	{1162., 3754.}	181.066	0.397153
46	newly 3 weeks spec	168.	337.545	{140., 678.}	108.214	0.278904	newly 3 weeks cumul	709.	1310.87	{573., 2447.}	91.6451	0.252781	newly 5 weeks spec	88.	197.836	{65., 380.}	134.153	0.30075	newly 5 weeks cumul	909.	1768.87	{753., 3362.}	101.508	0.268342	newly 51 weeks cumul	1016.	2304.73	{900., 4393.}	131.525	0.309206
47	newly 3 weeks spec	173.	300.271	{113., 573.}	91.9859	0.242022	newly 3 weeks cumul	651.	1201.59	{543., 2255.}	92.5851	0.239115	newly 5 weeks spec	105.	168.169	{59., 313.}	90.3148	0.245594	newly 5 weeks cumul	904.	1596.44	{695., 3012.}	89.3468	0.234532	newly 52 weeks cumul	1045.	2002.61	{819., 3861.}	102.838	0.24911
48	newly 3 weeks spec	241.	441.667	{249., 763.}	85.2401	0.234426	newly 3 weeks cumul	934.	1558.75	{927., 2475.}	68.1775	0.204048	newly 5 weeks spec	108.	296.571	{149., 508.}	174.603	0.384254	newly 5 weeks cumul	1207.	2219.6	{1258., 3623.}	84.3571	0.235588	newly 49 weeks cumul	1531.	3284.98	{1760., 5456.}	114.695	0.289729
49	newly 3 weeks spec	134.	326.377	{157., 543.}	143.565	0.325366	newly 3 weeks cumul	470.	1267.55	{644., 2157.}	169.692	0.385865	newly 5 weeks spec	57.	191.522	{74., 311.}	236.003	0.44381	newly 5 weeks cumul	619.	1709.04	{843., 2901.}	176.097	0.38929	newly 51 weeks cumul	792.	2260.84	{1042., 3850.}	185.46	0.393437
50	newly 3 weeks spec	82.	97.4754	{49., 160.}	47.0612	0.165753	newly 3 weeks cumul	351.	522.82	{262., 918.}	60.3475	0.171235	newly 5 weeks spec	30.	35.1148	{17., 55.}	48.9617	0.174946	newly 5 weeks cumul	410.	614.902	{314., 1086.}	61.0196	0.171748	newly 56 weeks cumul	413.	666.377	{341., 1169.}	69.4161	0.18738
